# Remnant of Unrelated Amniote Sex Chromosomal Linkage Sharing on the Same Chromosome in House Gecko Lizards, Providing a Better Understanding of the Ancestral Super-Sex Chromosome

**DOI:** 10.3390/cells10112969

**Published:** 2021-11-01

**Authors:** Worapong Singchat, Thitipong Panthum, Syed Farhan Ahmad, Sudarath Baicharoen, Narongrit Muangmai, Prateep Duengkae, Darren K. Griffin, Kornsorn Srikulnath

**Affiliations:** 1 Animal Genomics and Bioresource Research Center (AGB Research Center), Faculty of Science, Kasetsart University, 50 Ngamwongwan, Chatuchak, Bangkok 10900, Thailand; worapong.si@ku.th (W.S.); thitipong.pa@ku.th (T.P.); syedfarhan.a@ku.th (S.F.A.); 2 Laboratory of Animal Cytogenetics and Comparative Genomics (ACCG), Department of Genetics, Faculty of Science, Kasetsart University, 50 Ngamwongwan, Chatuchak, Bangkok 10900, Thailand; 3 Special Research Unit for Wildlife Genomics (SRUWG), Department of Forest Biology, Faculty of Forestry, Kasetsart University, 50 Ngamwongwan, Chatuchak, Bangkok 10900, Thailand; prateep.du@ku.ac.th; 4 Bureau of Conservation and Research, Zoological Park Organization of Thailand, Bangkok 10300, Thailand; sudarath.b246@gmail.com; 5 Department of Fishery Biology, Faculty of Fisheries, Kasetsart University, 50 Ngamwongwan, Chatuchak, Bangkok 10900, Thailand; ffisnrm@ku.ac.th; 6 School of Biosciences, University of Kent, Canterbury CT2 7NY, UK; d.k.griffin@kent.ac.uk; 7 Amphibian Research Center, Hiroshima University, 1-3-1 Kagamiyama, Higashihiroshima 739-8526, Japan

**Keywords:** chromosome map, gecko lizard, bacterial artificial chromosome, fusion, super-sex chromosome, evolution

## Abstract

Comparative chromosome maps investigating sex chromosomal linkage groups in amniotes and microsatellite repeat motifs of a male house gecko lizard (*Hemidactylus frenatus*, HFR) and a flat-tailed house gecko lizard (*H. platyurus*, HPL) of unknown sex were examined using 75 bacterial artificial chromosomes (BACs) from chicken and zebra finch genomes. No massive accumulations of microsatellite repeat motifs were found in either of the gecko lizards, but 10 out of 13 BACs mapped on HPL chromosomes were associated with other amniote sex chromosomes. Hybridization of the same BACs onto multiple different chromosome pairs suggested transitions to sex chromosomes across amniotes. No BAC hybridization signals were found on HFR chromosomes. However, HFR diverged from HPL about 30 million years ago, possibly due to intrachromosomal rearrangements occurring in the HFR lineage. By contrast, heterochromatin likely reshuffled patterns between HPL and HFR, as observed from C-positive heterochromatin distribution. Six out of ten BACs showed partial homology with squamate reptile chromosome 2 (SR2) and snake Z and/or W sex chromosomes. The gecko lizard showed shared unrelated sex chromosomal linkages—the remnants of a super-sex chromosome. A large ancestral super-sex chromosome showed a correlation between SR2 and snake W sex chromosomes.

## 1. Introduction

The number of de novo genome sequence assemblies (reference genomes) is increasing, particularly in amniote lineages, due to advanced technologies that generate high-quality long reads, greater read depths, and eventual assemblies with fewer and longer contigs per genome [1]. However, the ability to assemble a genome with the same number of contigs as chromosomes (‘chromosome level’ assembly) remains the goal of de novo sequencing. For example, the genome sequences of farm animals—chicken, pig, and cow—were assembled as physical maps before Sanger sequencing [2,3,4]. However, there is little next-generation sequencing data for a large number of species. There have been significant advances in novel techniques such as optical mapping (i.e., BioNano), long-read sequencing (PacBio and Oxford Nanopore Technologies (ONT)), Hi-C linkage mapping (Dovetail), and pre-existing chromosome-level reference assemblies [5,6,7]. Nevertheless, technical issues still exist and contigs cannot be assembled across centromeres or large heterochromatic blocks [8,9,10]. The fluorescence in situ hybridization (FISH) technique should therefore be applied to build a physical chromosome map in several map-poor species before starting the genome project, in order to investigate biological evolutionary constraints in genome structure [11,12,13]. It is also necessary to integrate technologies for whole-sequence data with molecular cytogenetics in so-called ‘chromosomics’ [14].

The gecko lizard (Squamata: Gekkota) is phylogenetically located at the base of squamate reptiles, excluding the Dibamidae, and estimated to have diverged from the common ancestor of non-dibamid squamates around 170–240 million years ago (MYA) [15,16,17,18]. A long evolutionary history has produced a species-rich and geographically widespread group of gecko lizards that currently includes approximately 2095 species [19]. Despite the great interest in squamate reptile chromosome evolution and diversity of sex determination in these species, chromosome maps (derived from molecular cytogenetics approaches) have been constructed for only a few gecko lizard species [20,21,22,23,24]. Chromosome numbers in gecko lizards vary from 2*n* = 16–46 [25,26], with most karyotypes composed of 38–42 chromosomes [27,28]. Along with the high interspecific homogeneity of most gecko lizard karyotypes, several intraspecific chromosomal polymorphisms were observed in *Gekko*, *Hemidactylus*, and *Tarentola* species [22,28,29,30]. Contrary to most non-avian reptile karyotypes with macro and microchromosomes, most gecko lizard chromosome complements consist of acrocentric elements (sometimes with a few metacentrics) that gradually decrease in size, as observed in lacertid lizards [23,31,32]. This suggests that the microchromosomes disappeared due to fusions between macro and microchromosomes, and/or between microchromosomes in the gecko lizard lineage [23,24]. However, karyotype evolution within the gecko lizard lineage is derived from centric fusion/fission and multiple pericentric inversions independently in each family and between species within the same family [20,22,23,24,27]. Therefore, it is believed that chromosomal rearrangements influence the genomic reorganization and function under position effects of the gecko lizard lineage [33,34,35].

In addition to different karyotypic features in gecko lizards, their sex determination systems (SDSs) exhibit large diversity [22,36]. The ancestor of gecko lizards is hypothesized to have had environmental-dependent sex determination (ESD) followed by frequently repeated transitions to both XX/XY and ZZ/ZW SDSs, with some species retaining the ancestral ESD [36,37,38,39]. Male-derived heterogametic systems with X_1_X_1_X_2_X_2_/X_1_X_2_Y neo-sex chromosomes are also found in pygopodids [40,41]. However, comparative chromosome FISH mapping shows scant evidence for linkage homology among sex chromosomes, whereas quantitative PCR (qPCR) identification might provide partial sex chromosomal linkage homology in the gecko lizard [23,42,43,44,45]. Gecko lizards appear to exhibit an unusually high frequency of turnovers in SDSs during their evolutionary process, creating variability in sex determination under the ‘evolutionary trap hypothesis’ [36,46]. Repeated turnovers of SDS in the gecko lizards provide opportunities to better understand the evolutionary forces and processes driving sex chromosome transitions [47]. Accordingly, gecko lizard sex determination is labile, and *Hemidactylus* exhibits intrageneric transitions. House gecko lizards (*Hemidactylus* spp.) include species with both XX/XY and ZZ/ZW sex chromosomes and ESD [22,36]. Thus, they are an excellent group for testing general hypotheses on sex determination and sex chromosome evolution. Interestingly, partial sex chromosomal linkage homologies have also been found between chromosomes in different amniote lineages, while unrelated sex chromosomes share linkage homologies across distantly related groups, such as the Hokou gecko (*Gekko hokouensis*, Pope, 1928) and chicken (*Gallus gallus*, Linnaeus, 1758) [8,9,10,21,24,48,49,50,51,52,53,54]. Currently, squamate reptile chromosome 2 (SR2) and snake W sex chromosomes are hypothesized to share partial sex chromosomal linkage homologies with sex-related elements or common genomic regions, such as repeats, supporting the concept that the two chromosomes were associated with a larger ancestral amniote super-sex chromosome before multiple fissions into different lineages [8,9,10,24,48,49,50,51,52,55,56].

Although this hypothesis has been studied reasonably well in various species, it is worth investigating the evolutionary context of unrelated sex chromosomal linkage homologies in the sex chromosome lineages of the gecko lizard. Their karyotype evolution likely evolved in the opposite direction, determined by identifying microchromosome repeated fusion, compared to several squamate reptiles [23,24]. Such a finding could improve our understanding of the transition mechanisms between different sex chromosome forms and raise the possibility of shared linkage homology through an ancestral super-sex chromosome. Given this scenario, together with the chromosome maps of several amniotes [8,9,10,21,23,24,32,57,58,59,60,61,62,63,64,65,66,67,68,69,70,71], we proposed two hypotheses: (1) *There is a shared unrelated sex chromosomal linkage between amniotes, as in the gecko lizards*, or (2) *no segment of shared unrelated sex chromosomal linkages of amniotes exists in gecko lizards*. To address these hypotheses, we constructed a comparative chromosome map of two house geckos (the common house gecko (*Hemidactylus frenatus*, HFR) (Duméril and Bibron, 1836) [72] and the flat-tailed house gecko (*Hemidactylus platyurus*, HPL) (Schneider, 1797) [73], considering sex chromosomal linkage groups in amniotes using FISH, with 75 BACs derived from the chicken and zebra finch genomes [8,9,10,74,75,76]. Karyotypes of the two house gecko lizards likely contain homomorphic sex chromosomes [19] and are expected to have fewer repeat elements on their sex chromosomes than species with heteromorphic sex chromosomes [77,78]. We compared linkage homology between these two house geckos and other amniotes using chromosomic technologies [8,9,10,21,23,24,32,62,63,64,65,66,67,68,69,70,71,79]. The resultant chromosome maps also allowed us to detect changes in positions when the order of BACs was modified due to chromosomal rearrangements. We discuss the possibility of shared unrelated linkage homologies through a postulated ancestral super-sex chromosome.

## 2. Materials and Methods

### 2.1. Specimen Collection, Cell Culture, Chromosome Preparation

A male house gecko lizard (*H. frenatus*: HFR) and a flat-tailed house gecko lizard (*H. platyurus*: HPL) of unknown sex were examined. Detailed information is presented in Table 1. Species identification was performed based on morphological characteristics [80,81]. Animal care and all experimental procedures were approved by the Animal Experiment Committee, Kasetsart University, Thailand (approval no. ACKU59-SCI-006) and conducted in accordance with the Regulations on Animal Experiments at Kasetsart University. The heart, lungs, and mesentery of the two house geckos were removed after intraperitoneal injection of pentobarbital, and used for cell culture. The sex of each animal was identified morphologically and confirmed from the internal anatomy. Tissues were minced and cultured in Dulbecco’s modified Eagle’s medium (Life Technologies—Gibco) supplemented with 15% fetal bovine serum (Life Technologies—Gibco), 100 μg/mL kanamycin, and 1% antibiotic-antimycotic (Life Technologies—Gibco). Primary cultured fibroblasts were incubated at 27 °C in a humidified atmosphere of 5% CO_2_ in air and sub-cultured using trypsin. Chromosome preparations were conducted using a standard air-drying method [62]. Fibroblasts at the logarithmic phase of the growth cycle were subjected to colcemid treatment (100 ng/mL) for 45 min and fixed (methanol:acetic acid, 3:1 per volume basis (*v*/*v*)) after hypotonic treatment in 0.075 M KCl 20 min before harvesting. Cell suspensions were dropped onto clean glass slides and air-dried. The slides were stored at −80 °C until required for analysis. The morphology and size of chromosomes were characterized and arranged according to Levan et al. [82], Turpin and Lejeune [83] and Trifonov et al. [22].

### 2.2. Meiotic Chromosome Preparation

Meiotic chromosome preparations for light microscopy were produced using the air-dry method described previously [62,84]. Briefly, after hypotonic treatment in 1% sodium citrate for 20 min at room temperature, the seminiferous tubules were placed in a fixative solution (1:1 ethanol/acetic acid) for 3–4 min, and then in a 60% fixative solution (1:1 fixative diluted with distilled water) for 3 min on ice. The mass of the seminiferous tubules was unraveled using dissecting needles, and swollen germ cells were then spread out from the ends of the tubules. The germ cells were collected by filtration using gauze and dissolved with 1:1 ethanol/acetic acid. Cells in suspension were dropped onto clean glass slides and air-dried. The slides were stained with 3% Giemsa in a phosphate buffer (pH 6.8) for 10 min.

### 2.3. C-Bandings

To examine the chromosomal distribution of constitutive heterochromatin, C-banding was performed using the standard barium hydroxide/saline/Giemsa method (Summer, 1972) [85] with slight modification as follows: chromosome slides were treated with 0.2 N HCl at room temperature for 60 min and then with 5% Ba(OH)_2_ at 50 °C for 1.30 s, followed by treatment with 2× SSC at 65 °C for 60 min.

### 2.4. Fluorescence In Situ Hybridization Mapping of Telomeric Repeat and Microsatellite Repeat Motifs

The chromosomal locations of telomeric (TTAGGG)_n_ sequences and 19 microsatellite repeat motifs: (CA)_15_, (GC)_15_, (GA)_15_, (AT)_15_, (CAA)_10_, (CAG)_10_, (CAT)_10_, (CGG)_10_, (GAG)_10_, (AAT)_10_, (AAGG)_8_, (AATC)_8_, (AGAT)_8_, (ACGC)_8_, (AAAT)_8_, (AAAC)_8_, (AATG)_8_, (AAATC)_6_ and (AAAAT)_6_ were determined using FISH, as described previously [8,62,86]. We used commercially biotin-labeled 42-bp oligonucleotide complementary to (TTAGGG)_n_ sequences and 19 commercially biotin-labeled oligonucleotide microsatellite repeat probes (Macrogen, Inc., Seoul, Korea), ethanol precipitated with salmon sperm DNA, and *Escherichia coli* tRNA. After hybridizing biotin-labeled probes to HFR and HPL chromosomes, the probes were detected by incubating the chromosome slides with avidin labeled with fluorescein isothiocyanate (avidin-FITC; Invitrogen, CA, USA). Slides were subsequently stained with 1 µg/mL DAPI (4′, 6′-diamidino-2-phenylindole). Fluorescence hybridization signals were captured using a cooled charge-coupled device (CCD) camera mounted on a Nikon ECLIPSE 80i microscope and processed using NIS-Elements software version 3.22 (Nikon Instruments Inc., Tokyo, Japan).

### 2.5. Isolation, Amplification and Labeling of Chicken and Zebra Finch BACs

Chicken and zebra finch BACs were used for cross-species FISH mapping based on the range of the proportion of conserved elements shared across multiple species. Given the high degree of apparent genome conservation between avian and reptilian species [8,9,10,74,75,76], these sets of BACs were applied to HFR and HPL. In total, 51 chicken and 24 zebra finch BACs were anchored to chicken and zebra finch genome assemblies by linkage and sequencing comprising chicken chromosome 1 (GGA1), GGA2p, GGA4p, GGA5, GGA6, GGA9, GGA13, GGA15, GGA17, GGA23, GGA27, GGA28 and chicken sex chromosome Z (GGAZ), and zebra finch chromosome 1 (TGU1B), TGU4A, TGU5, TGU6, TGU9, TGU13, TGU15, TGU17, TGU23, TGU27, TGU28 and TGUZ. The BAC clone DNA was isolated using a Qiagen Miniprep Kit (Qiagen, Manchester, UK) before amplification and direct labeling by nick translation (Roche, Welwyn Garden City, UK). Probes were labeled with Texas Red 12-dUTP (Invitrogen Corporation and Applied Biosystems Inc., Carlsbad, CA, USA) and fluorescein isothiocyanate (FITC)-12-UTP (Roche) before purification using the Qiagen Nucleotide Removal Kit (Qiagen).

### 2.6. Cross-Species BAC FISH Mapping

Chromosome slides were dehydrated through an ethanol series (2 min each in 2× SSC, 70%, 85% and 100% ethanol at room temperature). Probes were diluted in a hybridization solution (Cytocell Ltd., Cambridge, UK) with chicken hybloc (Insight Biotechnology Ltd., London, UK) and applied to HFR and HPL chromosomes on a 37 °C hotplate before being sealed with rubber cement. Probe and target DNA were denatured simultaneously on a 75 °C hotplate before hybridization for 2 min in a humidified chamber at 37 °C for 72 h. Slides were washed post-hybridization for 30 s in 2× SSC/0.05% Tween 20 at room temperature before being counterstained using VECTASHIELD antifade mounting medium with 4′,6-diamidino-2-phenylindole (DAPI; Vector Laboratories, Inc., Burlingame, CA, USA). Images were captured using an Olympus BX61 Epifluorescence Microscope with a cooled CCD camera and SmartCapture system (Digital Scientific UK Ltd., Cambridge, UK). Confirmation of BAC signal order was achieved by dual-color experiments, where Texas Red 12-dUTP- and FITC-12-UTP-labeled probes were hybridized simultaneously.

## 3. Results

**Figure 1:**
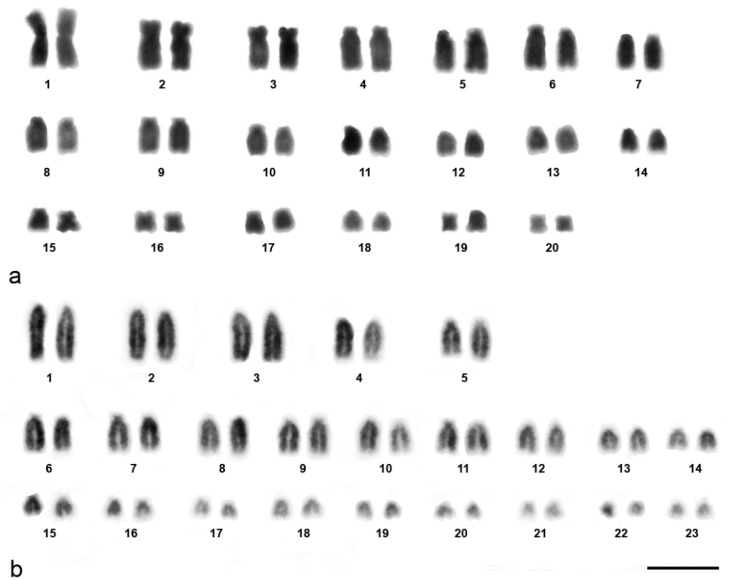
Gray images of DAPI-stained karyotypes of the common house gecko (*Hemidactylus frenatus*, Duméril and Bibron, 1836) [72] (**a**) and the flat-tailed house gecko (*Hemidactylus platyurus*, Schneider, 1797) [73] (**b**). Scale bar represents 10 μm.

We examined more than 20 DAPI-stained metaphase spreads for a male HFR and an HPL of unknown sex. The diploid chromosome numbers were 40 and 46 in HFR and HPL, respectively. In HFR, diploid chromosomes were exhibited as one large pair (HFR1) and four small pairs (HFR16, 17, 19, 20) of metacentric, two pairs of submetacentric (HFR2, 3), and the rest as acrocentric elements, while HPL comprised of 23 pairs of acrocentric elements of gradually decreasing size (Figure 1).

**Figure 2:**
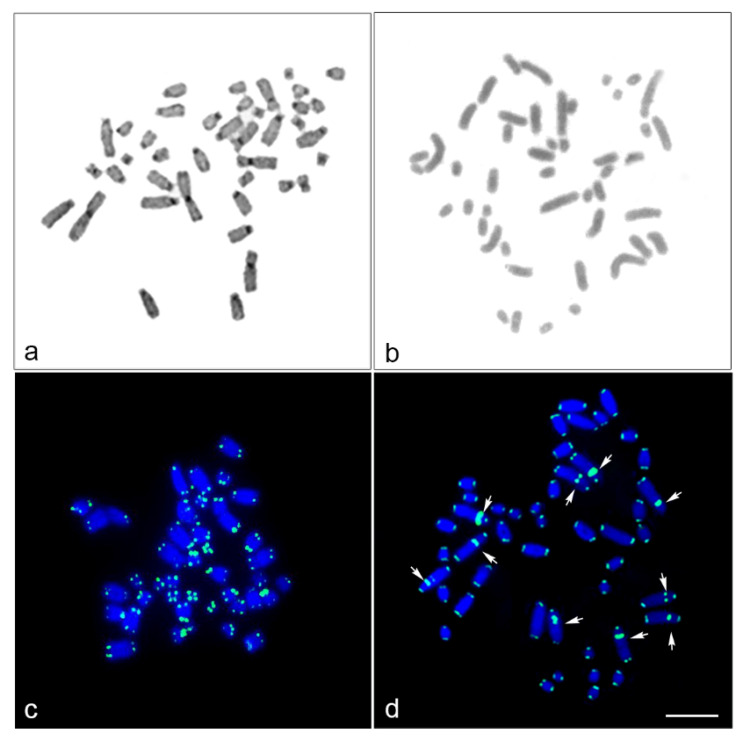
C-banded metaphase spread, and chromosomal locations of the telomeric (TTAGGG)_n_ sequence in the common house gecko (*Hemidactylus frenatus*, Duméril and Bibron, 1836) [72] and the flat-tailed house gecko (*Hemidactylus platyurus*, Schneider, 1797) [73]. C-banded metaphase spread in HFR (**a**) and HPL (**b**). FISH patterns of the telomeric (TTAGGG)_n_ sequence on DAPI-stained metaphase chromosome spreads of HFR (**c**) and HPL (**d**). Arrows indicate signals of interstitial telomeric sites. Scale bars represent 10 μm.

A DAPI-stained chromosome banding pattern was not found in HFR; DAPI-negative bands were contractually observed in the proximal region of the long arm of five large chromosome pairs in HPL1–3 and 5–6, indicating GC richness in this region. C-positive heterochromatin was observed in the centromeric region in each chromosome of HFR but not in HPL (Figure 2). Hybridization signals of hexamer repeat sequences (TTAGGG)_n_ were found at the ends of all chromosomes in both HFR and HPL (Figure 2).

**Figure 3:**
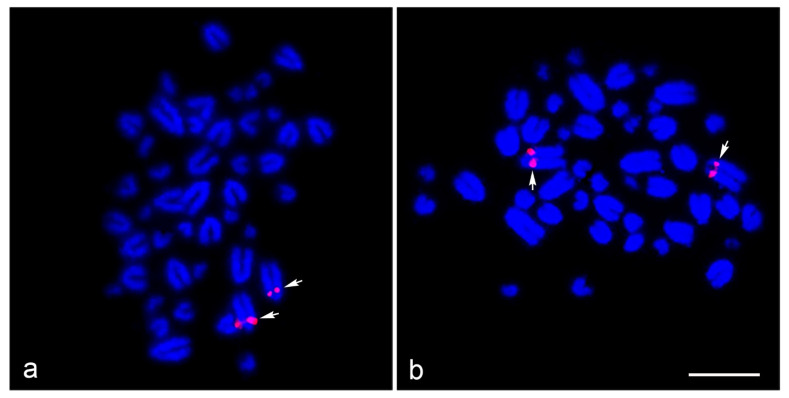
Chromosomal locations of chicken and zebra finch BACs of the flat-tailed house gecko (*Hemidactylus platyurus,* HPL) (Schneider, 1797) [73]. GGA23 BAC (Texas Red-labeled TGMCBA-272G9) was located on chromosome 1 (HPL1) (**a**) and GGA27 BAC (Texas Red-labeled CH261-28L10) was located on chromosome 4 (HPL1) (**b**). Arrows indicate hybridization signals. Scale bar represents 10 µm.

**Figure 4:**
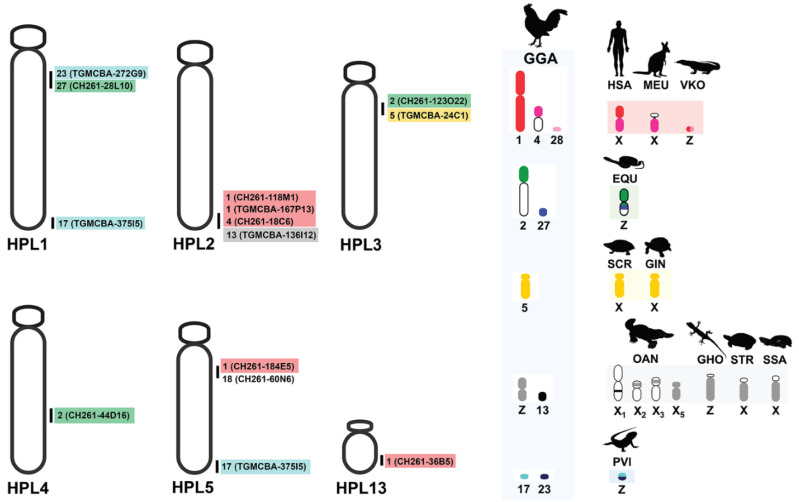
Chromosome map of the flat-tailed house gecko (*Hemidactylus platyurus*, HPL) (Schneider, 1797) [73], showing chromosome homologies with chicken and zebra finch. This map was constructed with 13 chicken and zebra finch BACs mapped on the flat-tailed house gecko chromosome 1–5 and chromosome 13. Locations of BACs are shown to the right of the flat-tailed house gecko chromosomes. The chromosome numbers show the chromosomes of the chicken (*Gallus gallus,* GGA) (Linnaeus, 1758) [54] and zebra finch (*Taeniopygia guttata*, TGU) (Vieillot, 1817) [91] homologous to the flat-tailed house gecko chromosomes, and chromosomes sharing homologies with sex chromosome of several other amniotes. Partial sex chromosomal linkage homologies are shown in the same color. Chromosomal locations of genes in the amniotes were obtained from comparative gene mapping (chromosome mapping via a cytogenetic technique) and whole genome sequencing as the following sources: (*Gallus gallus,* GGA) from Matsuda et al. [57], humans (*Homo sapiens*, HSA) and tammar wallaby (*Macropus eugenii*, MEU) from Grützner et al. [58], duck-billed platypus (*Ornithorhynchus anatinus*, OAN) from Veyrunes et al. [59], bearded dragon lizard (*Pogona vitticeps*, PVI) from Ezaz et al. [61], Hokou gecko (*Gekko hokouensis*, GHO) from Kawai et al. [21], Komodo dragon (*Varanus komodoensis*, VKO) from Lind et al. [65], Japanese four-striped rat snake (*Elaphe quadrivirgata*, EQU) from Matsubara et al. [66], marsh turtle (*Siebenrockiella crassicollis*, SCR) from Kawagoshi et al. [67], wood turtle (*Glyptemys insculpta*, GIN) and Mexican musk turtle (*Staurotypus triporcatus*, STR) from Montiel et al. [68], and giant musk turtle (*Staurotypus salvinii*, SSA) from Kawagoshi et al. [69].

Interstitial telomeric sites were found on the long arm of five large chromosome pairs in HPL1–3 and 5–6, with the same location of DAPI-negative bands. None of the 19 microsatellite repeat motifs were successfully mapped onto HFR and HPL chromosomes (data not shown). Light microscopy of the meiotic configuration in the spermatocytes of male HFR showed normal stages of meiosis (Figure S1). There were 20 bivalents in diakinesis-metaphase I (MI) for HFR, and 40 chromosomes in metaphase II (MII) for HFR as diploid species. Diakinesis-MI cells with partially paired bivalents speculated to be heteromorphic X and Y chromosomes, and MII cells with condensed chromosomes speculated to be the Y chromosome, were not detected. However, we could not observe any meiotic cells from the HPL because of its juvenile state. Different degrees of cross-species BAC hybridization reflect how conserved segments have changed during evolution (Table 2 and Supplementary Table S1) [8,9,10,74,75,76,87,88,89]. Here, chicken and zebra finch BACs located on GGA1, GGA2p, GGA4p, GGA5, GGA13, GGA17, GGA18, GGA23 and GGA27 were mapped to the HPL (eight chicken BACs and five zebra finch BACs; Figure 3, Figure 4, Figure S2 and Table 2).

No chicken and zebra finch BACs were contractually mapped in HFR chromosomes. More than 20 metaphase spreads were observed for each BAC, with hybridization efficiencies ranging from 70% to 90%. Chromosome homology among HPL, chicken, and zebra finch was analyzed using the chicken genome database (https://www.ncbi.nlm.nih.gov/genome/annotation_euk/Gallus_gallus/104/, accessed on 1 July 2020) and the zebra finch genome database (https://www.ncbi.nlm.nih.gov/genome/annotation_euk/Taeniopygia_guttata/103/, accessed on 1 July 2020). The thirteen BACs mapped on HPL chromosomes included three BACs mapped on HPL1 that were homologous to GGA17p (TGMCBA-375I5), GGA23 (TGMCBA-272G9), and GGA27q (CH261-28L10), four BACs mapped on HPL2 that were homologous to GGA1q (CH261-118M1), GGA1 (TGMCBA-167P13), GGA4 (CH261-18C6) and GGA13 (TGMCBA-136I12) and two BACs mapped on HPL3 that were localized to GGA2p (CH261-123O22) and GGA5 (TGMCBA-24C1). One BAC mapped on HPL4 was localized to GGA2q (CH261-44D16), three BACs mapped on HPL5 were homologous to GGA1q (CH261-184E5), GGA17p (TGMCBA-375I5) and GGA18p (CH261-60N6), and one BAC mapped on HPL13 was located on GGA1 (CH261-36B5) (Figure 3, Figure 4 and Table 2).

## 4. Discussion

In this study, we performed cross-species BAC FISH mapping of two house gecko lizards. Results showed that only 6.5% of the BACs provided identifiable signals attributable to the evolutionary distance between chicken or zebra finch and Gekkonidae of approximately 320 MYA [17,93], while we achieved a 23.5% success rate mapping chicken/zebra finch BACs in snakes [8,10], and 13.0% in Iguanidae [9], which diverged from chicken 90 and 125 MYA, respectively [93] (Table S1). This suggests that success rates of cross-species FISH with BACs decrease rapidly with increasing evolutionary distance. However, no specific hybridization signal was observed in HFR, although HFR diverged from HPL about 30 MYA [36]. This might be caused by a strong intrachromosomal rearrangement occurring in the HFR lineage. By contrast, heterochromatin likely formed a reshuffling pattern between HPL and HFR, as observed from C-positive heterochromatin distribution. This was also reflected by increased levels of non-specific background hybridization of repetitive elements in HFR. Predicting the genomic content of all BACs in this study revealed that most were LINE and SINE, in addition to the microsatellite repeat motifs [8,9,10]. The presence and absence of hybridization signals from mapped BACs in different species might result from the amplification of repetitive sequences that occur independently in each lineage and at different rates [74,75,76]. The same BACs were used to perform chromosome mapping in snakes showing large blocks of hybridization signals, probably derived from repetitive sequences; however, the same location of ITS and mapped BAC in HPL showed twin dots of hybridization signals in this study [8,9,10].

Despite SDS’ importance in numerous ecological and evolutionary processes in the gecko lizard lineage, they have not converged to a single SDS mechanism. Dynamics of their SDSs and karyotypic patterns lead us to propose that an extensive process of genomic reorganization has occurred in this lineage compared with other squamate reptiles [23,24,39,94,95]. When considering sex chromosomal linkage homologies and an ancestral amniote super-sex chromosome, SR2 and snake W sex chromosomes share partial sex chromosomal linkage homologies with sex-related elements or common genomic regions of other amniotes [8,9,10,24,48,50,51,52]. The stochastic occurrence of chromosomal rearrangements in the gecko lizard lineage is, thus, appropriate to test the current direction of an ancestral super-sex chromosome, as distinct from other squamate reptiles. Although the SDS of HPL remains unknown, cross-species comparative BAC mapping showed that 10 of 13 BACs mapped on HPL chromosomes associated with other sex chromosome amniotes (Table 2, Figure 3). Five BACs (GGA1q (CH261-118M1), GGA1q (CH261-184E5), GGA1 (CH261-36B5), GGA1 (TGMCBA-167P13), and GGA13 (TGMCBA-136I12)) showed partial homology with SR2 and one BAC (GGA1, TGMCBA-167P13) with snake Z and/or W sex chromosomes. Notably, a partial linkage homology of GGA23 appeared on the Z and W chromosomes of the bearded dragon (*Pogona vitticeps*, Ahl, 1926) [96,97]. Twin dots of hybridization signals of the BAC (TGMCBA-272G9) derived from GGA23 were mapped on HPL1 in the pericentromeric region. These were co-localized with the BAC (CH261-28L10) derived from GGA27. Based on the comparative genomics in the gecko lizards [22,23], HPL1 is likely homologous to chromosome 3 of Schlegel’s Japanese gecko (*Gekko japonicus*, Schlegel, 1836) [92]. These observations of hybridization patterns of all BACs mapped on HPL lead us to support previous reports of shared unrelated partial sex chromosomal linkages that were also observed in the gecko lizard and a large ancestral super-sex chromosome that correlated between SR2 and snake W sex chromosomes [8,9,10,24,48,50,51,52]. Common genomic elements such as repetitive sequences might result in hybridizing the same BACs onto multiple different chromosome pairs, suggesting the contribution of transitions to sex chromosomes across amniotes. In addition, several microsatellite repeat motifs, exhibiting a massive accumulation with chromosome mapping, show repetitive sequences shared between the sex chromosomes of several amniotes [8,9,41,50,98,99,100,101,102,103]. However, no massive accumulations of microsatellite repeat motifs were found in HPL or HFR. This might result from the influence of karyotypic reorganization in the lineage of the gecko lizards and the state of homomorphic chromosomes between males and females of both species, similar to pythons [104]. The question remains whether any genomic elements are shared among unrelated sex chromosomal linkages in amniotes, which drive sex chromosome differentiation after the chromosomal rearrangement in each lineage. The amniote super-sex chromosome might also have shared certain de novo sequences via accidental random homology that still remain unknown. To evaluate this further, complete sequences of many amniote sex chromosomes should be thoroughly explored to investigate any putative sex-linked orthologs. This can be achieved by overcoming the challenges and difficulties of assembling sex chromosomes through the availability of high-quality genome assemblies and annotation of amniotes.

The common ancestor of amniotes is currently considered to have ESD, while sex chromosomes evolved independently in different lineages [48,105,106,107]. Based on the phylogenetic reconstruction of SDSs in reptiles, GSD seems to be evolutionarily stable in the evolution trap [46]. Sufficient degeneration between the X and Y (or Z and W) is able to inhibit transitions or turnover of the SDS into different systems. ESD mechanisms among amniotes come from a common ancestor and will likely have a common mechanism [46]. This predicts that evolutionary amniote lineages phylogenetically separated by an ESD lineage should independently have non-homologous sex chromosomes, such as those found in mammals, birds, and several squamate reptiles [36,46,108,109]. However, it should be pointed out that not all homomorphic sex chromosomes, as cytogenetically defined, are similar at the DNA sequence level [54,110,111]. Contrary to the trap, the frequent turnover of SDSs, which are distributed in many gecko lizards, most amphibians and many fish species, can inhibit differentiation when newly derived sex chromosomes have not had time to degenerate [112,113,114,115,116,117,118,119,120,121]. An alternative explanation is that some amniote chromosomes may have evolved repeatedly into sex chromosomes [8,9,10,24,48,49,50,51,52]. Certain genome sections might tend to be recruited to function as sex chromosomes more often than other parts due to their content of genes or genomic elements involved in gonad differentiation. These form a basis for the evolution of sex-determining genes by their conversions into partial sex chromosomal linkage homologies [105,106,122]. During this time, chromosomal rearrangements fragmented the ancestral super-sex chromosome, but some of the emerged chromosomes still retained the sex chromosomal role in phylogenetically distant amniote lineages [48].

This might explain why ancestral GSD predicts that ESD evolved multiple times within amniotes and that ESD can be placed within a lineage that also contains GSD species. However, this does not explain why the same partial genomic regions of the super-sex chromosomes are associated with X/Y or Z/W sex chromosomes. Complicated discussions ensue as to whether the evolution of sex chromosomes from ESD to GSD is random or whether certain linkage homologies have a higher chance of becoming a part of sex chromosomes. Perhaps, a mixture of the two hypotheses may exist, separated by a polygenic sex determination (PSD) system, wherein several genes initiate the SDS in a particular species. PSD can occur by modifying existing sex chromosomes to create a novel functional sex chromosome at the same locus or by modifying autosomal loci in other regions of the genome to provide new inputs for regulating gonadal development [123,124]. This may promote fitness benefits as natural selection in the population, suggesting that PSD engenders an evolutionarily stable aspect. Another example is the spotted snow skink (*Niveoscincus ocellatus*, Gray, 1845) [125] that exhibits polymorphic SDSs. Sex is determined by male heterogamety (XY) in a highland population, whereas in a lowland population, the offspring sex ratio is influenced by temperature [126]. Analysis of sex-linked SNP loci revealed that recombination between the X and Y could help maintain a mixed system in the lowland population. By contrast, temperature and genetics interacted to determine sex via the presence of sex-reversed females [127]. This might also result in the interaction of several functional genes and regulators to create novel functions of SDS. This could be challenged by the future discovery of additional amniote SDSs or the addition of homology data that identify cryptic transitions that do not involve a change in heterogamety. Knowing sex chromosome homology is necessary for properly counting the number of transitions between SDSs in general. Future studies should supply further information on the homology of sex chromosomes based on gene content and the homology of SDSs based on knowledge of sex-determining genes in amniotes. This could be used to generate a more reliable test of contrasting hypotheses on the evolution of sex determination in this crucial vertebrate lineage. Explanations for the non-random fusion of certain genomic parts with sex chromosomes need to be clarified in the future.

## 5. Conclusions

Amniotes are well known for their diverse sex determination mechanisms and sex chromosomes that exhibit extraordinary variability as either homomorphic or heteromorphic structures [48,128]. Current evidence suggests that gecko lizards retain the remnants of unrelated shared sex chromosomal linkages from amniotes. Combining our data with previously published studies indicated that unrelated sex chromosomal linkage homologies in amniotes were shared with SR2 and snake W sex chromosomes. However, we emphasize that data enabling the testing of the homology of SDSs across the gecko lizard lineage and amniotes are still very scarce. Comparative genomics of sex chromosomes must be performed based on good quality genome assemblies to identify the sex chromosome gene content and test the homology of sex chromosomes in a wider phylogenetic spectrum in amniotes. Our results challenge the notion of the sex chromosome evolution hypothesis with evidence for the existence of a colorful myriad of situations and trajectories involving many interacting processes.

## Figures and Tables

**Table 1:** List of species, chromosome number, sex determination and collection sites.

Species	Chromosome Number	Sex Determination	Collection Locality	Number of Animals Used (Female + Male)	Reference
*Hemidactylus frenatus*	*2n* = 40	ZZ/ZW	Bangkok	1 + 0	[22,36,90]
*Hemidactylus platyurus*	*2n* = 46	-	Bangkok	1 unknown	[22]

**Table 2:** List of chicken and zebra finch BACs mapped to common house gecko (*Hemidactylus frenatus*, HFR) (Duméril and Bibron, 1836) [72] and flat-tailed house gecko (*Hemidactylus platyurus*, HPL) (Schneider, 1797) [73] chromosomes and their chromosomal location in chicken (*Gallus gallus*, GGA) (Linnaeus, 1758) [54] and Schlegel’s Japanese gecko (*Gekko japonicus*, GJA) (Schlegel, 1836) [92].

GGA	Name	Result		GJA
		HFR	HPL	
1q	CH261-107E2	-	-	
1q	CH261-118M1	-	2	4
1q	CH261-168O17	-	-	
1q	CH261-184E5	-	5	2
1q	CH261-18J16	-	-	
1q	CH261-58K12	-	-	
1	CH261-36B5	-	13	2p, 11, 13
1	TGMCBA-167P13	-	2	4
1p	CH261-89C18	-	-	
1q	CH261-98G4	-	-	
2p	CH261-123O22	-	3	1q
2p	CH261-177K1	-	-	
2p	CH261-169N6	-	-	
2q	CH261-44D16	-	4	8
4	CH261-18C6	-	2	4
4	CH261-71L6	-	-	
4p	CH261-83E1	-	-	
4q	CH261-89P6	-	-	
5	CH261-122F8	-	-	
5	CH261-2I23	-	-	
5p	CH261-49B22	-	-	
5q	CH261-78F13	-	-	
5	TGMCBA-145C6	-	-	
5	TGMCBA-24C1	-	3	1q
6q	CH261-49F3	-	-	
6p	TGMCBA-382J4	-	-	
9p	CH261-183N19	-	-	
9q	CH261-187M16	-	-	
9	CH261-68O18	-	-	
9	CH261-95N3	-	-	
9	TGMCBA-150E19	-	-	
9	TGMCBA-217A3	-	-	
9	TGMCBA-321L6	-	-	
13p	CH261-115I12	-	-	
13	CH261-11H24	-	-	
13	CH261-59M8	-	-	
13	TGMCBA-136I12	-	2	4
13	TGMCBA-266O5	-	-	
13q	TGMCBA-321B13	-	-	
15	CH261-40D6	-	-	
15	CH261-48M1	-	-	
15p	CH261-90P23	-	-	
15	TGMCBA-231D20	-	-	
15q	TGMCBA-266G23	-	-	
17	CH261-113A7	-	-	
17q	CH261-42P16	-	-	
17	CH261-69M11	-	-	
17	TGMCBA-185B22	-	-	
17	TGMCBA-197G19	-	-	
17p	TGMCBA-375I5	-	1 and 5	3 and 2q
17	TGMCBA-67H23	-	-	
18p	CH261-60N6		5	2q
18q	CH261-72B18			
23	CH261-105P1	-	-	
23p	CH261-191G17	-	-	
23	CH261-49G9	-	-	
23q	CH261-90K11	-	-	
23	TGMCBA-173N15	-	-	
23	TGMCBA-227A15	-	-	
23	TGMCBA-272G9	-	1	3
23	TGMCBA-48O8	-	-	
27	CH261-100E5	-	-	
27q	CH261-28L10	-	1	3
27p	CH261-66M16	-	-	
27	TGMCBA-23C5	-	-	
27	TGMCBA-324P4	-	-	
28	CH261-101C8	-	-	
28	CH261-186C5	-	-	
28p	CH261-64A15	-	-	
28q	CH261-72A10	-	-	
Zp	CH261-129A16	-	-	
Zq	CH261-133M4	-	-	
Z	CH261-137F19	-	-	
Z	TGMCBA-200J22	-	-	
Z	TGMCBA-270I9	-	-	

-: No signal.

## Data Availability

Not applicable.

## Supplementary Materials

The following are available online at https://www.mdpi.com/article/10.3390/cells10112969/s1, Figure S1: Meiotic cell division of the common house gecko (*Hemidactylus frenatus*, HFR) (Duméril and Bibron, 1836). (a) interphase, (b) leptotene, (c) zygotene, (d) pachytene, (e) diplotene (f) diakinesis, (g) metaphase I, (h) metaphase II, (i) telophase I, and (j) telophase I. Scale bar represents 10 µm. Figure S2: Chromosomal locations of chicken and zebra finch BACs of the flat-tailed house gecko (*Hemidactylus platyurus*, HPL) (Schneider, 1797). GGA1 BAC (Texas Red-labeled CH261-36B5) was located on chromosome 13 (HPL13) (a), GGA1 BAC (Texas Red-labeled CH261-118M1) was located on HPL2 (b), GGA1 BAC (Texas Red-labeled CH261-184E5) was located on HPL5 (c), GGA1 BAC (Texas Red-labeled TGMCBA-167P13) was located on HPL2 (d), GGA2 BAC (Texas Red-labeled CH261-44D16) was located on HPL4 (e), GGA2 BAC (Fluorescein isothiocyanate (FITC)-labeled CH261-123O22) was located on HPL3 (f), GGA4 BAC (Texas Red-labeled CH261-18C6) was located on HPL2 (g), GGA5 BAC (Texas Red-labeled TGMCBA-24C1) was located on HPL3 (h), GGA13 BAC (FITC-labeled TGMCBA-136I12) was located on HPL2 (i), GGA17 BAC (FITC-labeled TGMCBA-375I5) was located on HPL1 and HPL5 (j), and GGA18 BAC (FITC-labeled CH261-60N6) was located on HPL5 (k). Arrows indicate hybridization signals. Scale bar represents 10 µm. Table S1: List of chicken and zebra finch BACs mapped to the common house gecko (*Hemidactylus frenatus*, HFR) (Duméril and Bibron, 1836) and the flat-tailed house gecko (*Hemidactylus platyurus*, HPL) (Schneider, 1797) chromosomes, compared with Siamese cobra (*Naja kaouthia*, NKA) (Lesson, 1797), Russell’s viper (*Daboia russelii*, DRU) (Shaw and Nodder, 1797), the common tiger snake (*Notechis scutatus*, NSC) (Peters, 1861), green iguana (*Iguana iguana*, IIG) (Linnaeus, 1758), common garden lizard (*Calotes versicolor*, CVE) (Daudin, 1802), water monitor lizard (*Varanus salvator macromaculatus*, VSA) (Deraniyagala, 1944), with their chromosomal location in chicken (*Gallus gallus*, GGA) (Linnaeus, 1758).
