# Supplementary material for: Remnant of Unrelated Amniote Sex Chromosomal Linkage Sharing on the Same Chromosome in House Gecko Lizards, Providing a Better Understanding of the Ancestral Super-Sex Chromosome
Source: Cells. 2021 Nov 1;10(11):2969. doi: 10.3390/cells10112969 (PMC8616239; doi:10.3390/cells10112969)
Supplement: Supplementary file 1 [file cells-10-02969-s001.zip › Table S1 V1.pdf]

Table S1 List of chicken and zebra finch BACs mapped to the common house gecko (*Hemidactylus frenatus*, HFR) (Duméril and Bibron, 1836) and the flat-tailed house gecko (*Hemidactylus platyurus*, HPL) (Schneider, 1797) chromosomes, compared with Siamese cobra (*Naja kaouthia*, NKA) (Lesson, 1797), Russell's viper (*Daboia russelii*, DRU) (Shaw and Nodder, 1797), the common tiger snake (*Notechis scutatus*, NSC) (Peters, 1861), green iguana (*Iguana iguana*, IIG) (Linnaeus, 1758), common garden lizard (*Calotes versicolor*, CVE) (Daudin, 1802), water monitor lizard (*Varanus salvator macromaculatus*, VSA) (Deraniyagala, 1944), with their chromosomal location in chicken (*Gallus gallus*, GGA) (Linnaeus, 1758).

Name	GGA	NKA	NSC	DRU	IIG	CVE	VSA	HFR	HPL
CH261-107E2	1q	-	-	-	-	-	-	-	-
CH261-118M1	1q	-	-	-	-	3	-	-	2
CH261-168O17	1q	-	-	-	-	-	-	-	-
CH261-184E5	1q	micro	-	-	-	3	-	-	5
CH261-18J16	1q	micro	-	-	-	-	micro	-	-
CH261-58K12	1q	-	-	-	-	-	-	-	-
CH261-36B5	1	micro	-	-	-	-	-	-	13
TGMCBA-167P13	1	Wq	W	W	-	-	-	-	2
CH261-89C18	1p	Wq	-	-	-	-	-	-	-
CH261-98G4	1q	-	-	W	-	-	5	-	-
CH261-123O22	2p	-	Z	-	6	-	4	-	3
CH261-177K1	2p	Z	-	-	-	-	-	-	-
CH261-169N6	2p	-	-	-	-	-	4	-	-
CH261-44D16	2q	-	3	3	-	4	7	-	4
CH261-18C6	4	1q and micro	-	-	-	-	-	-	2
CH261-71L6	4	Wq	-	W	-	-	-	-	-
CH261-83E1	4p	-	-	-	-	-	-	-	-
CH261-89P6	4q	-	-	-	-	-	-	-	-
CH261-122F8	5	-	1q	1q	-	1	-	-	-
CH261-2I23	5	1q	-	-	-	1	2q	-	-
CH261-49B22	5p	1q and micro	-	-	-	-	-	-	-
CH261-78F13	5q	-	-	-	-	-	2q	-	-

TGMCBA-145C6	5	1q	-	-	-	1	4	-	-
TGMCBA-24C1	5	-	-	-	-	1	-	-	3
CH261-49F3	6q	-	-	-	3	-	-	-	-
TGMCBA-382J4	6p	-	-	-	3	-	-	-	-
CH261-183N19	9p	micro	-	-	-	-	-	-	-
CH261-187M16	9q	micro	-	-	-	-	micro	-	-
CH261-68O18	9	-	-	-	-	3	-	-	-
CH261-95N3	9	Wq	6	6, W	-	3	-	-	-
TGMCBA-150E19	9	-	-	-	3	3	-	-	-
TGMCBA-217A3	9	-	-	-	-	-	-	-	-
TGMCBA-321L6	9	-	-	W	3	-	-	-	-
CH261-115I12	13p	-	-	-	-	-	-	-	-
CH261-11H24	13	-	-	-	micro?	-	1q	-	-
CH261-59M8	13	-	-	-	-	2q	-	-	-
TGMCBA-136I12	13	-	-	-	-	2q	1q	-	2
TGMCBA-266O5	13	-	-	-	2q	-	1q	-	-
TGMCBA-321B13	13q	-	-	-	2q	2q	-	-	-
CH261-40D6	15	micro	micro	-	-	2 and 3	-	-	-
CH261-48M1	15	-	micro	-	micro	micro	-	-	-
CH261-90P23	15p	micro	-	-	micro	micro	-	-	-
TGMCBA-231D20	15	-	-	-	-	-	-	-	-
TGMCBA-266G23	15q	micro	-	-	-	-	-	-	-
CH261-113A7	17	-	-	-	-	-	-	-	-
CH261-42P16	17q	-	-	-	-	-	-	-	-
CH261-69M11	17	micro	-	-	-	-	-	-	-
TGMCBA-185B22	17	-	-	-	-	-	-	-	-
TGMCBA-197G19	17	-	-	-	-	-	-	-	-
TGMCBA-375I5	17p	Wq	W	W	-	micro	-	-	1 and 5
TGMCBA-67H23	17	Wq	W	W	-	-	-	-	-
CH261-60N6	18p	-	-	-	-	-	-	-	5
CH261-72B18	18q	-	-	-	-	-	-	-	-
CH261-105P1	23	micro	-	-	-	-	-	-	-

CH261-191G17	23p	micro	-	-	-	-	-	-	-
CH261-49G9	23	micro	-	-	-	-	-	-	-
CH261-90K11	23q	-	micro	-	-	micro	-	-	-
TGMCBA-173N15	23	micro	-	-	-	-	-	-	-
TGMCBA-227A15	23	Wq	W	-	-	-	-	-	-
TGMCBA-272G9	23	-	-	-	-	-	-	-	1
TGMCBA-48O8	23	micro	-	-	-	-	-	-	-
CH261-100E5	27	-	-	-	-	6	-	-	-
CH261-28L10	27q	-	-	-	-	-	4	-	1
CH261-66M16	27p	Z	-	-	-	-	4	-	-
TGMCBA-23C5	27	Zq and Wq	W	-	-	-	-	-	-
TGMCBA-324P4	27	-	-	-	-	-	-	-	-
CH261-101C8	28	-	-	-	-	-	-	-	-
CH261-186C5	28	-	-	-	-	-	-	-	-
CH261-64A15	28p	micro	-	-	-	-	-	-	-
CH261-72A10	28q	micro	-	-	-	-	-	-	-
CH261-129A16	Zp	Wq	-	-	micro?	-	1p	-	-
CH261-133M4	Zq	2q	2p, W	-	2p	2p	1p	-	-
CH261-137F19	Z	-	-	-	-	-	-	-	-
TGMCBA-200J22	Z	Wq	W	-	-	-	-	-	-
TGMCBA-270I9	Z	2q	W	W	-	-	1p	-	-
