# Supplementary figures and images for: Remnant of Unrelated Amniote Sex Chromosomal Linkage Sharing on the Same Chromosome in House Gecko Lizards, Providing a Better Understanding of the Ancestral Super-Sex Chromosome
Source: Cells. 2021 Nov 1;10(11):2969. doi: 10.3390/cells10112969 (PMC8616239; doi:10.3390/cells10112969)

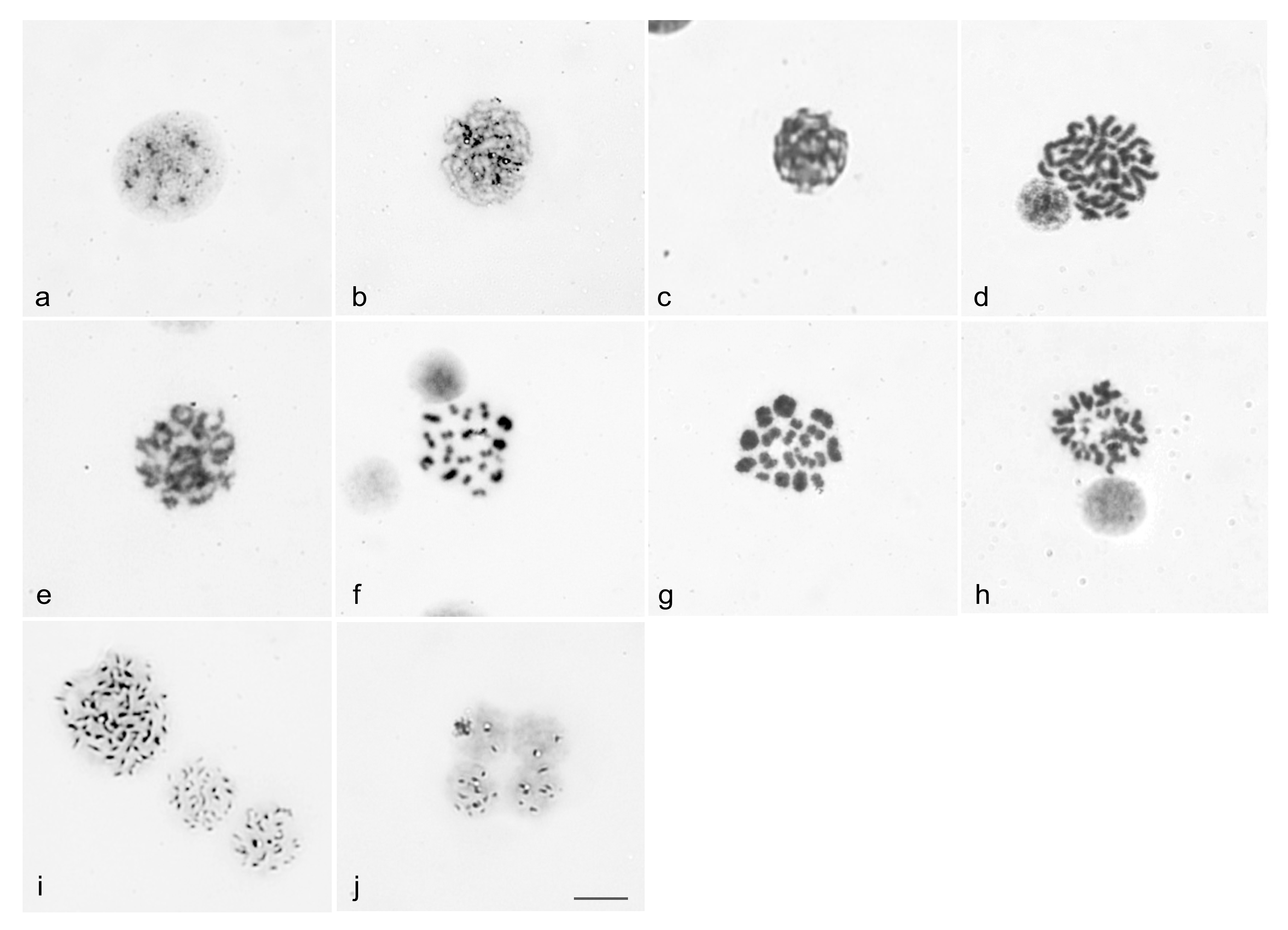

Supplement: Supplementary file 1 [file cells-10-02969-s001.zip › Figure S1 meiotic V2.tif]

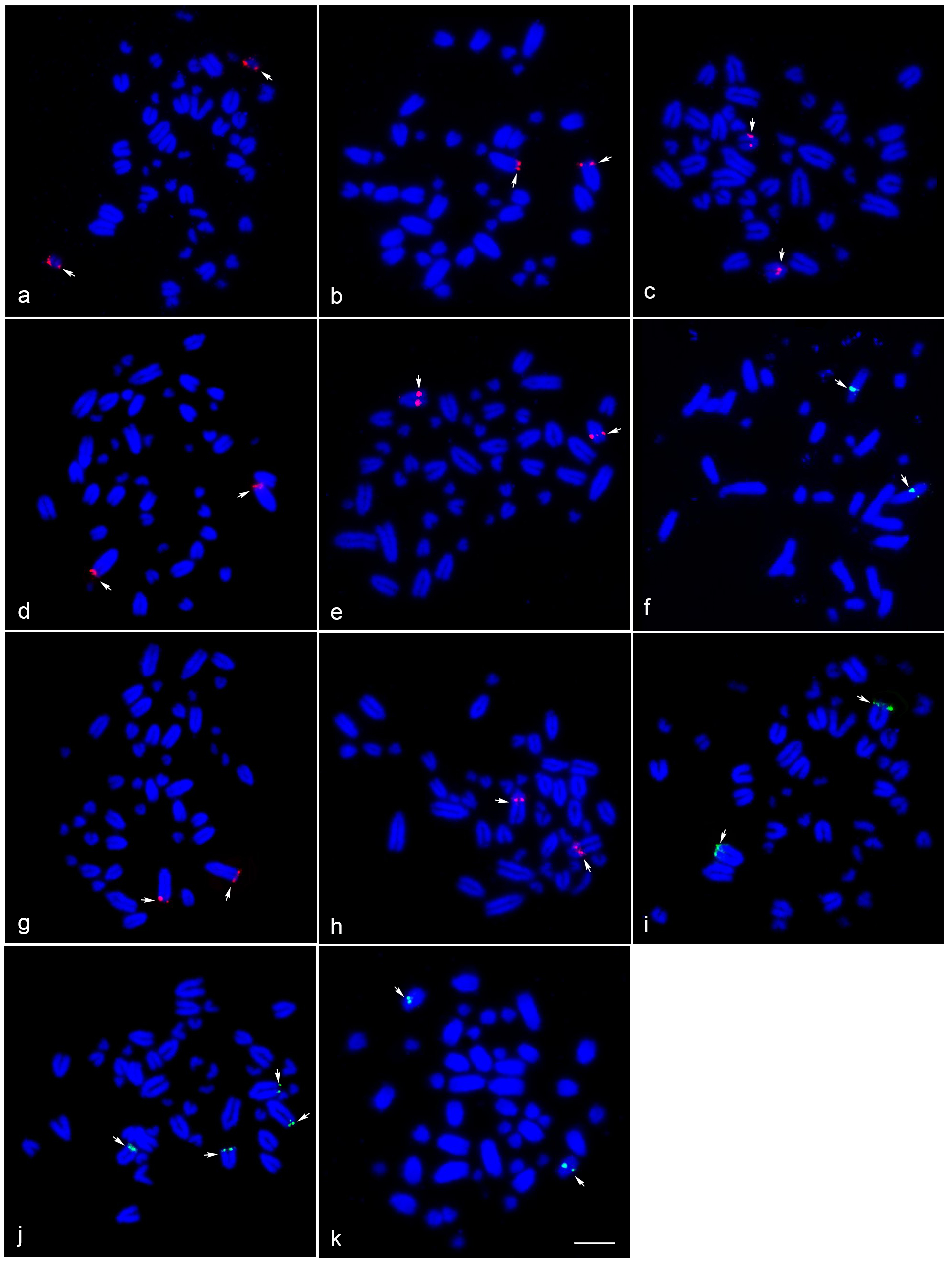

Supplement: Supplementary file 1 [file cells-10-02969-s001.zip › Figure S2 other chromosome map results V2.tif]
